# Remarks upon Cerebro-Spinal Meningitis, with Reports of Cases

**Published:** 1873-08-01

**Authors:** J. H. Hollister

**Affiliations:** Chicago Medical College


### REMARKS UPON CEREBRO-SPINAL MENINGITIS, WITH REPORTS OF CASES.

BY PROF. J. H. HOLLISTER, OF CHICAGO MEDICAL COLLEGE.

While the above name will readily designate the disease to which I wish to refer, I am sure many observers will agree with me that the term does not by any means compass its pathology.

It is also obvious, that while we may easily state what we do *not* know with reference to it, it is a matter *not so easy* to state much of which we *are* positive.

It *is* certain that it is often one of the gravest diseases which we are called to treat, and quickly decisive in results.

It *is* certain that, here and there, it appears among the people in endemic form, and yet giving no possible clue to the reason why any particular person should become its victim.

It *is* certain, also, that in some localities it has the prevalence and characteristics of an epidemic.

Some of its pathological phenomena are also clearly expressed, while doubtless others equally important are as yet undiscovered; nor is it now possible to argue *a priori* as to when, and where, and under what circumstances it shall prevail, and where and under what circumstances there shall be exemption.

It is by no means certain that the producing cause holds any intimate relation with that malaria which genders periodical fevers. A thousand instances can be cited where intermittent diseases have prevailed extensively, with not a case of meningitis. Neither is it true that it does not appear in our large cities. Cases without number are found in the geographical centers of our largest cities—cases where no malarial symptoms were manifest, either in the person, or to be found, so far as could be ascertained, in the entire neighborhood, and where the patient had been for a long period of time subject only to such influences as were developed in their own homes.

It will not do, therefore, hastily to assume its relations and dependence upon what we term malaria, any more than it will do to assume, with our present knowledge, that the tawny complexion of those who live in malarious districts is dependent upon the debris of the dying "ague plant."

Nor can we, I think, assume that any given numbers of symptoms are invariable factors of this disease.

Whatever the producing cause may be, one thing mid many *uncertainties is* certain, and that is, that, as it is developed in different persons, it has widely different manifestations. Time will not permit me now to enumerate them; but in this connection I will submit *two* carefully noted cases, which, so far as they go, will corroborate the above:

CASE I. — That of a robust little boy, aged four years. His home, on Michigan avenue, in Chicago, gave him no proclivity to ague. His health had been unusually good; he was, in fact, the picture of health and happiness. No imprudence in eating or exposure could explain to us the cause of the attack.

June 21st, 1872, was called to see him. Complaining of occasional pain a little below the stomach, with slight sensation of cramping of the abdominal muscles, and vomiting at intervals. The bowels had recently moved naturally; the tongue showed no appreciable derangement of the secretions; the mind was perfectly clear in its action; and the circulation was as yet undisturbed.

I sought, by one-grain powders of calomel, given at intervals, followed by a portion of oil, to gently move the bowels, and relieve from the effects of any disturbing ingesta—for as yet I did not anticipate the development of this disease.

June 22d. — There had been a moderately free movement of the bowels; and though it had been followed by an anodyne mixture, the pain in the abdomen was still more intense, and the vomiting more severe, As yet the pulse was scarcely disturbed, and there were *no* cerebro-spinal symptoms to be detected.

It seemed to me, after careful examination, that I had to do with some form of ganglionic nervous affection, and that the solar plexus was its location. Hoping to allay the morbid excitability, I prescribed five-grain doses of the bromide of potash every four hours, and at the intervening two hours a mixture representing in each dose three drops of chloroform and three of deodorized tincture of opium.

June 23. — The general symptoms were not only not relieved, but, in connection with the abdominal symptoms, there was clonic spasmodic action of the lumbar muscles; and now, for the first time, there was cervical opisthotinos. The patient yet replied intelligently, but now began to complain of pain in the head.

June 24. — There was rigidity of the posterior cervical muscles, with difficulty of deglutition; muttering delirium; slight strabismus; constant tendency to rotation of head to left side, with but little increase in the force or frequence of the pulse, and no vomiting. The action of the bowels, accomplished by enemas, gave no unhealthy appearance, and during this period the kidneys performed their functions naturally. The urine was natural in color, and contained no albumen.

The tincture of calibar bean, with fluid extract of ergot, was now given as freely as the condition of the patient seemed to warrant.

June 25. — The cervical opisthotinos was permanent; the right upper and lower extremities were as unmoved as if perfectly paralyzed; the left hand was carried to the nose with great uniformity of action, as often as twice every minute for hours at a time, and as often brought back to fall passively at the side; the partial rotation of the head to the right side was as uniformly continuous; the pupils were largely dilated, and did not respond to the stimulus of light. This general condition of things continued, without obvious diminution, till the fatal termination on the 30th of June. As the vital powers began to fail the pulse became gradually accelerated, but there was no special depression at any time, and no marked febrile action. During the last two days the opisthotinos was not so marked, and the use of the right hand and foot were regained, showing that there had been no permanent paralysis of that side.

There was at no time the mottled appearance which has given to this disease the name of "Spotted Fever." The question will arise: Was this a genuine case of Cerebro-Spinal Meningitis? I reply that, during the last ten years, I have seen many cases, with and without the spotted appearance, and I had not a doubt of its nature; and this opinion was fully substantiated by several of our leading physicians, and among them the senior editor of The Examiner, who was several times with me in consultation. There was no opportunity in this case for *post mortem* examination. We may note in this case the absence of febrile action; the absence of any cutaneous capillary congestion; and the absence for two days and a half, at the outset, of any symptoms which could be referred to the *head.* No pain was located there; the facial expression was natural; the pupils responded to light; the mental powers seemed undisturbed, and the cranial nerves seemed for that period to perform their functions naturally. The natural condition of the neck, and the steady action of the heart and respiration forbade the idea that either the pneumogastric or the spinal nerves were involved in centric *lesion.*

Case II. — Annie McCammon, living in the geographical center of Chicago, with no malarial disease affecting persons in the vicinity; aged twelve years; rather frail of form and delicate, and possessed of a very impressible nervous organization. Had enjoyed her usual health, and arose, feeling fully as well as usual, on Sabbath morning, May 11th, when, without any assignable cause, at eight o'clock, she commenced vomiting, at the same time complaining of severe headache.

Almost immediately, with violent spasmodic action, she became nearly unconscious, after several hours, answering a sharp loud inquiry in a monosyllable. Opisthotinos was at once developed; the head was so thrown back that swallowing was difficult; the pupils nearly equaled the iris in size; respirations 20 per minute, pulse 80.

May 12.—Lying quiet. Opisthotinos continued; difficulty in arousing her: complains then of pain in the head; pupils slightly sensitive to light; kidneys acting naturally; bowels quiet.

May 13.—Still complains of headache, and refers it to the frontal region; pupils still dilated; skin cool; no fever; pulse 70 per minute, and soft; muscular contraction the same; deglutition very difficult; respirations natural; bowels quiet; urine free; answers a little more freely.

May 14.—Muscular contraction still as severe; urine loaded with phosphates; no action of bowels; deglutition difficult; sleeps a little, but very restless; pulse *only* 46 per minute; respirations 14.

May 15.—Semi-comatose: can be roused to answer briefly; pulse 62; respirations 16, and nearly natural; opisthotinos still severe; motion of the head occasions great pain; skin warm, dry, and of a slightly pinkish hue; urinates freely and consciously; pain in the head severe; pupils nearly natural: respond readily to light; strabismus noticed for the first time; congestion of conjunctiva; restless during the night.

May 16.—Lies nearly unconscious; pulse 64; respirations a little labored; groans constantly with pain in the head; head still drawn back with great force; pupils of unequal size, and irregular in shape; answers imperfectly; swallows with less difficulty; urine free, with sediment; bowels moved *twice*, and naturally; skin warm, and still of delicate pinkish hue; tongue moist and natural, and has been from the first.

May 17.—Opisthotinos a little diminished; consciousness considerably restored; groans with each breath; still complains of great pain in the head; pupils nearly the natural size; some thirst; pulse 72, and nearly natural; skin warm, but no feverish feeling; *no rash at any time;* no discoloration; had one sleep of two hours' duration; average sleep half an hour.

May 18.—More conscious; eyes natural; complains of pain in the head piteously; extreme *thirst;* bowels incline to diarrhœa; urine pretty free; skin warm—no discoloration; pulse only 52 per minute, and feeble; prostration *more marked.*

*Treatment to date.*—At the onset, I procured what I knew to be a reliable tincture of calabar bean, and an equally reliable fluid extract of ergot. I so combined them that each teaspoonful represented, with the ergot, fifteen drops of the tincture of calibar bean. I ordered a teaspoonful to be given every four hours; and for these six days no other medicine had been administered. I now ordered one grain of quinine to be given in a teaspoonful of syrup of phosphates, at equal intervals two hours after, and alternating with the first prescription.

May 19.—Consciousness restored; pulse 60; respirations natural; calls incessantly for *food;* not so thirsty; opisthotinos a little relieved; diarrhœa controlled with turpentine emulsions; urine scanty, but natural; arms pained by moving; skin cool and natural; no rash; sight double.

May 20.—More natural in appearance; pulse 70, and natural; opisthotinos still persistent; constantly complaining of pain in the head. Continued quinine and phosphates, with half the dose of ergot and calabar bean.

May 21.—Symptoms worse; pulse 72, and soft; skin soft and natural; severe opisthotinos; clonic spasms of the entire post spinal muscles; pupils again largely dilated; seeming paralysis of left side; constant carrying to or retiring the right hand from the head; tendency to prostration more marked; stupor; urine free; bowels quiet. Continued quinine and phosphates, and ordered full doses of ergot and calabar bean, and six drops tinct. opii. deodorata, with each dose of the last.

May 22.—Lies nearly stupid; symptoms same as yesterday. Same treatment continued.

May 23.—Lies nearly as if she were in sleep; aroused with difficulty, but answers correctly; pupils better contracted; pulse 60 and feeble; skin warm and natural; *no pain.* Continued same treatment, omitting the opium.

May 24.—Very conscious; pupils again natural, and contract readily to light; skin cool; extremeties cold; pulse 60, and feeble; very marked pain in the abdomen, in the region of the solar plexus; pain in the head again severe, "Oh, my head!" being for hours the almost momentary ejaculation; less pain in the use of the arms; bowels quiet; kidneys acting naturally.

May 25. — Consciousness fully restored; pulse 72, and normal; respirations and temperature natural; opisthotinos *less;* pupils natural, and sensitive to light; no paralysis; no action of the bowels; relishes food; still has the constant outcry of pain in the head; no other pain.

May 26. — General symptoms much improved; pulse 72, rather feeble, but otherwise natural; vision clear and distinct; deglutition easy; opisthotinos has nearly disappeared; no complaint of pain; skin natural; urine free; bowels quiet; appetite good.

May 26. — Steadily convalescing; prostration severe; emaciation very marked; opisthotinos entirely relieved; pulse and respipirations natural; pupils a little dilated; appetite good; bowels still unmoved.

May 27. — Symptoms as yesterday; gradual improvement.

May 28. — Still improving; free movement of the bowels; no pain; mind clear, and *very* active.

May 31. — Three days later: steadily improving; symptoms all favorable; pulse and respirations natural; secretions natural; pupils still a little dilated; the head aching a little; mind unusually active; appetite good.

I discontinued the ergot and calabar on the 26th inst., and the quinine and phosphates on the 30th. Good nutrition was constantly administered, *from first to last.* The best of prepared essence of beef was administered as often and as regularly as her medicines were, and in very liberal quantities. She took of the tincture of calabar bean and extract of ergot, at regular intervals of four hours, in quantity as stated, for fourteen days; and if a less amount were given, the pupils were again dilated, and the evidences of cerebral-capillary congestion most manifest.

I hazarded the issue upon these articles; and the result justified what may seem their heroic use.

My notes are fragmentary, and wanting in methodical arrangement; but I have chosen to transcribe them just as each day they were penciled at the bedside, not having drawn on my memory for a single statement of symptoms; so that, if not symmetrical, the description is in every point reliable.

I note here: 1st, An absence, entirely, of febrile action. 2d, There are none of the results of meningial inflammation as sequences of the disease. 3d, There was no cutaneous capillary congestion, by which it might be designated "Spotted Fever." 4th, if there be one manifestation more constant than the others, I think it is the intense cervical opisthotinos; and this points to a local effect upon the medulla, not only at the origin of the sub-occipital, but also of the spinal accessory nerves, by the stimulation of which such contraction is accomplished, and so long maintained. 5th, What we now need, is an extended series of carefully recorded facts, made at the time, and at the place, and covering all important points. From these it may be safe at length to generalise and theorise, perhaps, but not now.

Finally: If, as hitherto, my friends are prone to rely, as I have done, upon calabar and ergot, I counsel that they obtain preparations upon which they can implicitly rely, and test them to the full.

